# Switch rates, time-to-switch, and switch patterns of antiretroviral therapy in people living with human immunodeficiency virus in Japan, in a hospital-claim database

**DOI:** 10.1186/s12879-019-4129-6

**Published:** 2019-06-10

**Authors:** Daniel J. Ruzicka, Naho Kuroishi, Nobuyuki Oshima, Ryuta Sakuma, Toshio Naito

**Affiliations:** 1 Medical Affairs, MSD K.K, Kitanomaru Square, 1-13-12 Kudan-kita, Chiyoda-ku, Tokyo, 102-8667 Japan; 2 Japan Development, MSD K.K, Kitanomaru Square, 1-13-12 Kudan-kita, Chiyoda-ku, Tokyo, 102-8667 Japan; 3 Faculty of Medicine, Juntendo University, 2-1-1 Hongo, Bunkyo-ku, Tokyo, 113-0033 Japan

**Keywords:** HIV, Antiretroviral therapy, Switch rate, Time-to-switch, Switching pattern

## Abstract

**Background:**

Regardless of chronic treatment with antiretroviral therapy (ART), the switching rate for ART regarding anchor drugs has not been articulated in real-world clinical-settings in Japan. We assessed switch rates and time-to-switch of ART regimens according to anchor drug classes (integrase strand transfer inhibitors (INSTI), non-nucleoside reverse transcriptase inhibitors (NNRTI) and protease inhibitors (PI)) and common switching patterns of anchor drug classes in people living with human immunodeficiency virus (HIV) (PLWH) from 2008 to 2016.

**Methods:**

This retrospective, observational study used data of 1694 PLWH drawn from a large-scale medical claims database. The median time-to-switch and switch rates of anchor drug class were estimated by Kaplan-Meier analysis. To estimate 95% confidence intervals for switch rates and median days, the Brookmeyer and Crowley method and Greenwood method were used respectively. The switching patterns were summarized based on the time of switching. The switch rates were compared between two anchor drug classes for each year using log-rank tests.

**Results:**

We focused our results on 2011–2016 (*n* = 1613), during which most ART prescriptions were observed. A total of 268 patients switched anchor drug class from the first to a second regimen. The switch rate constantly increased over four years for NNRTIs (17.8–45.2%) and PIs (16.2–47.6%), with median time-to-switch of 1507 and 1567 days, respectively, while INSTI maintained a low switch rate (2.3–7.6%), precluding median-days calculation. The majority originally treated with NNRTI and PI switched to INSTI regardless of the switching timing after starting the first regimen (< 1 year: 91.7 and 97.5%, respectively, and ≥ 1 year: 100.0 and 97.5%, respectively). The risk of switching anchor drug classes was lower for INSTI than for other anchor drug classes in the first regimen even after adjusting for potential confounding factors.

**Conclusions:**

Patients with an ART regimen including INSTI as an anchor drug class maintained a low switch rate for long durations. The major switching strategies of anchor drug class for secondary treatment were from NNRTI or PI to INSTI. These results suggest that INSTI may be a durable anchor drug class for PLWH on ART although there are limitations inherent to the database.

**Electronic supplementary material:**

The online version of this article (10.1186/s12879-019-4129-6) contains supplementary material, which is available to authorized users.

## Background

Antiretroviral therapy (ART) has increased the survival of people living with human immunodeficiency virus (HIV) (PLWH), resulting in an improved life expectancy [1–3] nearly the same as that of people without HIV [4, 5]. Owing to the need of chronic treatment with antiretroviral drugs, a prescription of a durable, simple ART regimen is key to alleviating the burden of lifelong treatment and to improve quality of life in PLWH.

When initiating ART, the latest guidelines recommend a combination regimen mostly consisting of two nucleoside reverse transcriptase inhibitors (NRTIs) as backbone plus a third drug (the anchor drug) from the other classes, including non-nucleoside reverse transcriptase inhibitors (NNRTIs), protease inhibitors (PIs), and integrase strand transfer inhibitors (INSTIs) [6, 7]. In Japan, five classes of antiretroviral drugs, NRTIs, NNRTIs, PIs, INSTIs, and entry inhibitors (EIs), are currently available [7]. Among the various available combination patterns, the doctor’s choice of an anchor drug plays an important role in the treatment strategy due to the limited number of backbone choices. To date, the evidence is scarce regarding the selection of the anchor drug for the initial ART.

During the lifelong treatment of HIV, PLWH commonly experience switches of ART regimens. The reasons for switching regimens can be varied including drug-related adverse events (AEs), viral suppression, drug toxicity, new drug availability, and simplification of a current regimen [8–10]. The launch of a new drug may also trigger regimen switches if physicians or PLWH are unsatisfied with the current regimen. Despite the frequent treatment modifications expected in the lifelong course of ART, the switching strategies have also not been clearly defined.

As the switching rate for ART with regards to anchor drugs has not been articulated, it is important to understand when and how the ART regimens are switched and to identify anchor drug classes that might be administered for longer durations in real-world clinical-settings in Japan. Therefore, the objective of the present study was to assess the switch rates and time-to-switch of ART regimens according to anchor drug class and to identify common switching patterns of anchor drug class in PLWH, using a large-scale Japanese medical claims database.

## Methods

### Study design and data source

This was a retrospective, observational database study. Data were extracted from a hospital-based medical claims database in Japan that was constructed by Medical Data Vision Co., Ltd. (MDV, Tokyo, Japan). As of August 2016, the MDV database contained patient-level information and claims data, including diagnoses, medical procedures, prescriptions, and hospitalization, of over 15 million patients in 270 hospitals in Japan. These hospitals can provide advanced treatments and include advanced treatment hospitals, general hospitals, and acute care hospitals that provide the combination of diagnosis and per-diem payment system.

### Study population

PLWH on an ART regimen were the target of interest. Our analyzed dataset included patients aged ≥18 years with a diagnosis record of HIV, B20–24 according to the International Statistical Classification of Diseases and Related Health Problems 10th Revision (ICD-10) codes and with a prescription record of ART between April 2008 and December 2016 (study period). A prescription record of ART was identified when at least one anchor drug in any of the following antiretroviral drug classes was prescribed during the study period: NNRTIs, PIs, INSTIs, and EIs. Patients with a code of HIV-2 infection were excluded.

### Outcomes

The primary outcomes were the switch rates and time-to-switch of ART regimens according to the anchor drug classes. The secondary outcomes were the switch rates and time-to-switch of anchor drug class-based ART regimen by type of backbone, characteristics of patients who experienced an anchor drug switch in each anchor drug class-based ART regimen, and common switching patterns of anchor drug class.

### Definitions

The extracted data included demographic patient characteristics (age, sex), year of first ART record in the database, prescription records of anchor drugs and backbone, comorbidities, history of hospitalization, and AIDS-defining illnesses.

#### Anchor drugs and backbones

The anchor drugs of the ART regimens were identified using receipt codes and classified into four classes (anchor drug classes) according to the anti-HIV drug classification available in Japan [7], as follows: 1) NNRTIs, 2) PIs, 3) INSTIs, and 4) EIs.

The backbones of the ART regimens were identified using receipt codes and classified into three categories: 1) tenofovir disoproxil fumarate (TDF), 2) abacavir (ABC), and 3) others. As tenofovir alafenamide fumarate (TAF)/emtricitabine (FTC) were not available before December 2016 in Japan, TAF/FTC was not included as a backbone in the present study.

#### ART regimen switch and time-to-switch

The ART regimen switch focused solely on anchor drug classes and was defined based on a switch in the anchor drug class used in the ART regimens. The time-to-switch of ART regimen was defined as the period from the date of the first record of anchor drug class in the ART regimen within patient data (index date) (defined as the first regimen) to the date of switch to another anchor drug class in the ART regimen (defined as second regimen) during the study period. The date of anchor drug class switch was defined as the date of prescription of the new anchor drug class within 28 days after the termination of the preceding ART regimen. When no initiation of any new anchor drug class was identified within 28 days after termination of the preceding ART regimen, the regimen was considered discontinued. A change of anchor drug within the same anchor drug class was not considered a switch.

#### AIDS-defining illnesses

AIDS-defining illnesses were identified if any of the following records were present in the month of the index date: HIV non-tuberculous mycobacteria, HIV cytomegalovirus infection, HIV candidiasis, HIV *Pneumocystis carinii* pneumonia, HIV Kaposi’s sarcoma, HIV Burkitt’s lymphoma, HIV non-Hodgkin’s lymphoma, HIV encephalopathy, HIV-associated dementia, slim disease, acquired immune deficiency syndrome, AIDS, neonatal HIV infection, and AIDS-related complex. The corresponding ICD-10 codes were listed in Table 1.

**Table 1:** ICD-10 codes used to identify AIDS-defining illnesses and comorbidities

Disease name	ICD-10 code
AIDS-defining illnesses	
HIV non-tuberculous mycobacteria	B20.0
HIV cytomegalovirus infection	B20.2
HIV candidiasis	B20.4
HIV *Pneumocystis carinii* pneumonia	B20.6
HIV Kaposi’s sarcoma	B21.0
HIV Burkitt’s lymphoma	B21.1
HIV non-Hodgkin’s lymphoma	B21.2
HIV encephalopathy	B22.0
HIV-associated dementia	B22.0
Slim disease	B22.2
Acquired immune deficiency syndrome	B24
AIDS	B24
Neonatal HIV infection	B24
AIDS-related complex	B24
Comorbidities	
HIV-related diseases	–
Cytomegaloviral disease	B25, B45, B59, C46.9, C81
Hypertension	I10, I12, I14–15
Dyslipidemia	–
Hypercholesterolemia or hyperlipidemia	E78.0–78.5
Hepatitis B/C coinfection	B18
Diabetes	–
Type 2 diabetes	E11–14
Bone disorder	–
Osteoporosis	M80–81
Vascular disease	–
Myocardial infarction	I21–22
Stroke	I64
Angina pectoris	I20
Hypertensive heart and renal diseases	I11, I13
Psychiatric disorders	–
Mania and depression	F30–32
Anxiety	F40–41
Psychosis	F20–29
Dementia	F01, F03
Insomnia	F51
Kidney disease	–
Chronic renal failure	N18–19
Urolithiasis	N20–21
Malignancy	C00–97

*ICD-10* International Statistical Classification of Diseases and Related Health Problems 10th Revision

#### Comorbidities

Comorbidities were identified if any of the following ICD-10 codes were present in the same month as the index date: HIV-related diseases, hypertension, dyslipidemia, hepatitis B/C coinfection, diabetes, bone disorder, vascular disease, psychiatric disorders, kidney disease, and malignancy. The corresponding ICD-10 codes were listed in Table 1.

#### History of hospitalization

A history of hospitalization was identified if a record of hospitalization was present during the ART regimen prescribed on the index date.

### Statistical analysis

The proportions of anchor drug class and backbones prescribed on the index date were obtained by year. The demographic and clinical characteristics of all patients on ART regimens were analyzed descriptively according to anchor drug class prescribed on the index date.

The median time-to-switch and switch rates according to anchor drug class prescribed on the index date and those stratified by the backbone were estimated using Kaplan-Meier analysis. For the estimate of 95% confidence interval (CIs), the Brookmeyer and Crowley method was used for median days and the Greenwood method was used for switch rates. The switch rates were compared between two anchor drug classes for each year using log-rank tests. Discontinuation of the regimen or regimen continuation to the end of study period were censored.

The demographic and clinical characteristics of patients who switched anchor drug classes in their ART regimens were analyzed descriptively according to the anchor drug class prescribed on the index date. The timings (< 1 and ≥ 1 year) were descriptively analyzed in patients who switched anchor drug class in their ART regimen according to the anchor drug class prescribed on the index date and the corresponding 95% CI using Wilson scores.

To identify potential confounding factors for switching ART regimens and the factors interacting with the anchor drug class, a multivariate Cox regression model was initially used for variable selection with a cut-off of *p* < 0.05 for the main effect and *p* < 0.10 for the interaction term. The time-to-switch was included as an objective variable and the anchor drug class, risk factors, and interaction term between anchor drug class and each risk factor were included as explanatory variables. If the interaction term had a *p* < 0.10, the variable was retained in the model even if the main effect of the variable had a *p* ≥ 0.05. Subsequently, the hazard ratio (HR) of each anchor drug class was calculated adjusting for remaining variables and stratified by interaction factors to estimate the risk of switching anchor drug classes from the ART regimen prescribed on the index date.

All statistical analyses were performed using SAS release 9.4 (SAS Institute Inc., Cary, NC, USA). The remaining statistical tests were two-sided with a significance level of 0.05.

## Results

### Patient disposition

**Fig. 1:**
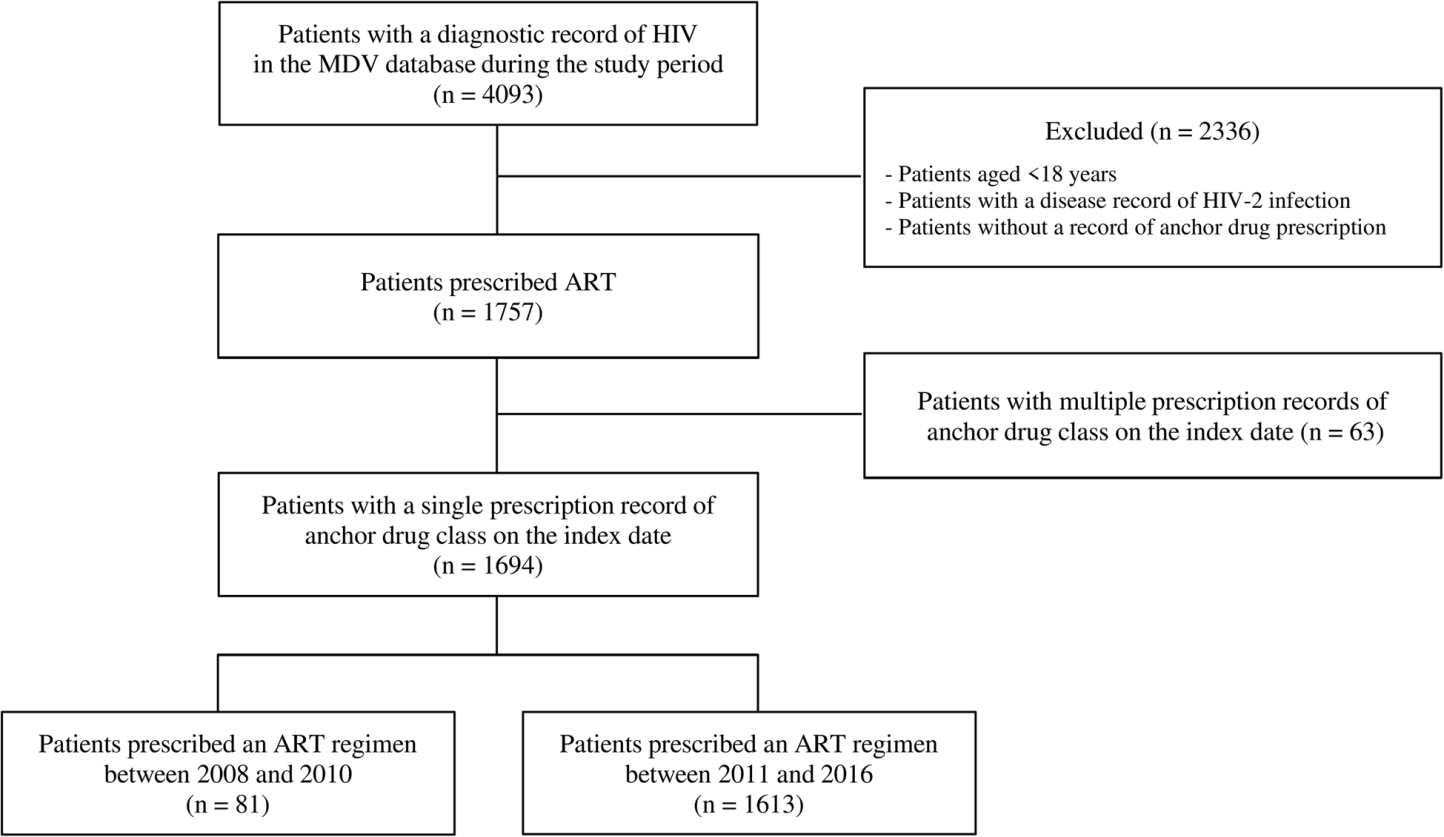
Patient dispositions. ART: antiretroviral therapy, HIV: human immunodeficiency virus

The patient disposition is shown in Fig. 1. A total of 4093 patients in the MDV database had a diagnostic record of HIV during the study period. Of those, 1757 were prescribed ART. After excluding 63 patients who were prescribed multiple anchor drug classes on the index date, 1694 patients had a single prescription record of anchor drug class on the index date. Broad usage of INSTI class in the database started after 2010 (first INSTI introduction in Japan was July 2008) and over 95% of the patients started an ART regimen between 2011 and 2016 (*n* = 1613) [Fig. 1], the main results reported in the present study focused on this time period.

### Distributions of anchor drug class and backbone in the ART regimens by year

**Fig. 2:**
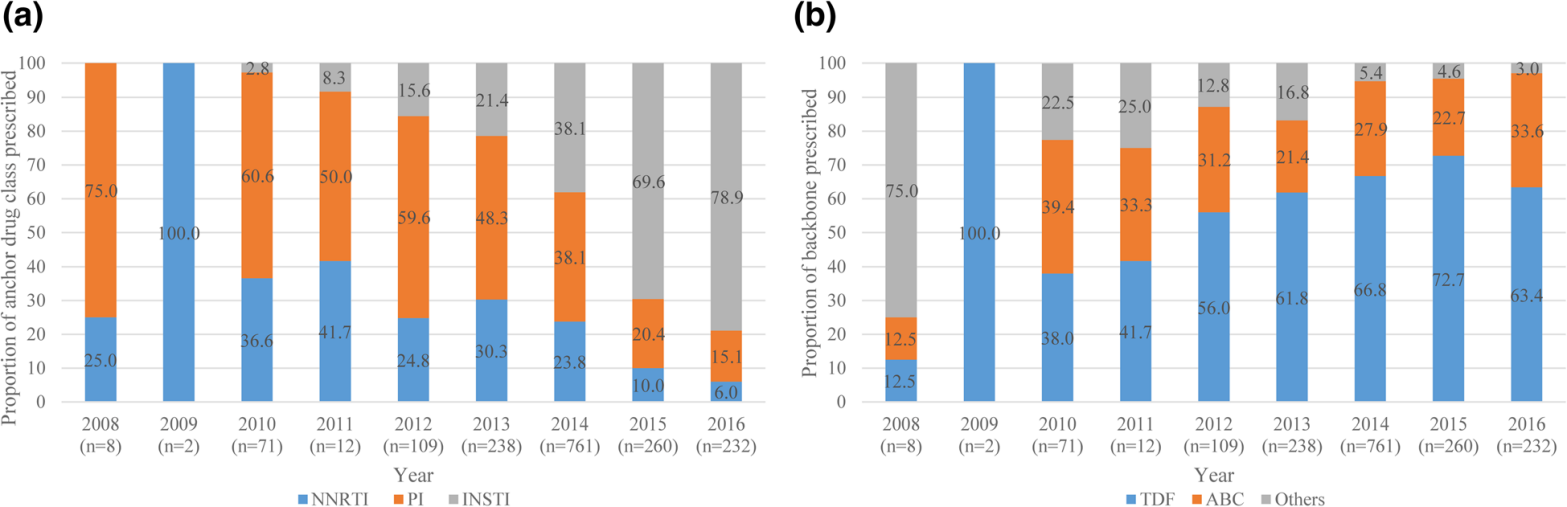
(**a**) Anchor drug classes and (**b**) backbones prescribed in first ART regimen (*n* = 1694). ART: antiretroviral therapy, NNRTI: non-nucleoside reverse transcriptase inhibitor, PI: protease inhibitor, INSTI: integrase strand transfer inhibitor, ABC: abacavir, TDF: tenofovir disoproxil fumarate, 3TC: lamivudine, FTC: emtricitabine. The years of launch of the anchor drugs by class are as follows: NNRTI: etravirine (launched in 2009) and rilpivirine (2012); PI: darunavir (2013); and INSTI: raltegravir (2008), dolutegravir (DTG) (2014), DTG/ABC/3TC (2015) and elvitegravir/cobi/TDF/FTC (2013). One patient prescribed an entry inhibitor in combination with others as a backbone in 2014 was excluded

The prescription of NNRTI and PI as anchor drug class in the ART regimen overall decreased after 2010 or 2011 (NNRTI: 41.7–6.0% between 2011 and 2016; PI: 60.6–15.1% between 2010 and 2016) [Fig. 2(a)]. Contrarily, the prescription of INSTI had increased rapidly since 2010 and accounted for over three-fourths of anchor drug classes prescribed in 2016 (2.8% in 2010 and 78.9% in 2016). Only one patient was prescribed EI as anchor drug class in 2014. Among backbones, TDF has been the predominant backbone since 2011 (41.7–72.7% between 2011 and 2016), followed by ABC (33.3% in 2011 to 33.6% in 2016) [Fig. 2(b)]. Others between 2011 and 2016 included mostly zidovudine (AZT)/lamivudine (3TC) accounted for 73.7% (AZT/3TC: 37.3% or 3TC and AZT: 36.4%) [see Additional file 1].

In 1613 patients who started an ART regimen during 2011–2016, 20.1% were on NNRTI, 35.0% on PI, and 44.8% on INSTI [Table 2]. Only one patient (0.1%) was prescribed an ART regimen with EI. Therefore, our results focused on NNRTI, PI, and INSTI. The mean (± standard deviation, SD) age of patients on INSTI was numerically younger (41.9 ± 11.9 years) and smaller proportions of patients had AIDS-defining illnesses (38.7%), dyslipidemia (18.5%), and diabetes (18.7%), compared to those in patients on NNRTI and PI (48.0 ± 12.5 and 45.4 ± 12.3 years; 47.4 and 46.6%; 29.8 and 26.8%; and 26.2 and 23.2%; respectively).

**Table 2:** Patient characteristics according to anchor drug class from 2011 to 2016 (*n* = 1613)

Characteristics	NNRTI (*n* = 325)	PI (*n* = 564)	INSTI (*n* = 723)
Age (years)			
Mean ± SD	48.0 ± 12.5	45.4 ± 12.3	41.9 ± 11.9
Range	20–82	22–90	18–82
Age group (years)			
18–39	89 (27.4)	188 (33.3)	324 (44.8)
40–59	166 (51.1)	291 (51.6)	324 (44.8)
≥ 60	70 (21.5)	85 (15.1)	75 (10.4)
Gender, male	299 (92.0)	488 (86.5)	683 (94.5)
AIDS-defining illnesses	154 (47.4)	263 (46.6)	280 (38.7)
HIV-related diseases	79 (24.3)	144 (25.5)	242 (33.5)
Hypertension	56 (17.2)	70 (12.4)	65 (9.0)
Dyslipidemia	97 (29.8)	151 (26.8)	134 (18.5)
Hepatitis B/C coinfection	48 (14.8)	69 (12.2)	103 (14.2)
Diabetes	85 (26.2)	131 (23.2)	135 (18.7)
Bone disorder	10 (3.1)	19 (3.4)	17 (2.4)
Vascular disease	8 (2.5)	13 (2.3)	15 (2.1)
Psychiatric disorders	29 (8.9)	61 (10.8)	84 (11.6)
Kidney disease	19 (5.8)	25 (4.4)	26 (3.6)
Malignancy	12 (3.7)	27 (4.8)	40 (5.5)
History of hospitalization	43 (13.2)	95 (16.8)	177 (24.5)
Year of ART initiation			
2011	5 (1.5)	6 (1.1)	1 (0.1)
2012	27 (8.3)	65 (11.5)	17 (2.4)
2013	72 (22.2)	115 (20.4)	51 (7.1)
2014	181 (55.7)	290 (51.4)	290 (40.1)
2015	26 (8.0)	53 (9.4)	181 (25.0)
2016	14 (4.3)	35 (6.2)	183 (25.3)

*NNRTI* non-nucleoside reverse transcriptase inhibitor, *PI* protease inhibitor, *INSTI* integrase strand transfer inhibitor, *SD* standard deviation, *HIV* human immunodeficiency virus, *ART* antiretroviral therapy

Values are expressed as number (percentage) unless specified otherwise

One patient prescribed an entry inhibitor was excluded from the table

#### Characteristics of patients who switched anchor drug class

Of all patients who started an ART regimen, 270 (16.7%) switched anchor drug classes. For each anchor drug class, 26.2% switched from NNRTI (85/325), 28.7% from PI (162/564), and 3.0% from INSTI (22/723) [Table 3]. Overall, no major differences were observed in characteristics among patients treated with these three predominant anchor drug classes. AIDS-defining illnesses accounted for 59.1% of patients originally treated with INSTI, while the proportions were lower in those originally treated with NNRTI or PI (45.9 and 40.7%, respectively). The proportion of patients with dyslipidemia was higher among those originally treated with NNRTI (35.3%) and INSTI (31.8%) than that among those treated with PI (25.3%). The proportion of patients with diabetes was highest among patients originally treated with NNRTI (30.6%), followed by those with PI (22.2%) and INSTI (13.6%).

**Table 3:** Characteristics of patients switching an anchor drug class from the first ART regimen from 2011 to 2016

Characteristics	Anchor drug class prescribed in the first ART regimen		
	NNRTI (*n* = 85)	PI (*n* = 162)	INSTI (*n* = 22)
Age (years)			
Mean ± SD	48.6 ± 11.4	43.6 ± 11.8	44.7 ± 13.4
Range	24–75	23–81	27–76
Age group (years)			
18–39	21 (24.7)	63 (38.9)	10 (45.5)
40–59	46 (54.1)	79 (48.8)	8 (36.4)
≥ 60	18 (21.2)	20 (12.3)	4 (18.2)
Gender, male	81 (95.3)	146 (90.1)	19 (86.4)
AIDS-defining illnesses	39 (45.9)	66 (40.7)	13 (59.1)
HIV-related diseases	22 (25.9)	33 (20.4)	6 (27.3)
Hypertension	17 (20.0)	23 (14.2)	4 (18.2)
Dyslipidemia	30 (35.3)	41 (25.3)	7 (31.8)
Hepatitis B/C coinfection	11 (12.9)	19 (11.7)	3 (13.6)
Diabetes	26 (30.6)	36 (22.2)	3 (13.6)
Bone disorder	3 (3.5)	4 (2.5)	1 (4.5)
Vascular disease	3 (3.5)	3 (1.9)	1 (4.5)
Psychiatric disorders	11 (12.9)	20 (12.3)	2 (9.1)
Kidney disease	8 (9.4)	8 (4.9)	1 (4.5)
Malignancy	2 (2.4)	9 (5.6)	1 (4.5)
History of hospitalization	11 (12.9)	26 (16.0)	9 (40.9)
Year of ART initiation			
2011	–	1 (0.6)	–
2012	10 (11.8)	19 (11.7)	–
2013	14 (16.5)	28 (17.3)	3 (13.6)
2014	59 (69.4)	99 (61.1)	15 (68.2)
2015	1 (1.2)	9 (5.6)	2 (9.1)
2016	1 (1.2)	6 (3.7)	2 (9.1)

*ART* antiretroviral therapy, *NNRTI* non-nucleoside reverse transcriptase inhibitor, *PI* protease inhibitor, *INSTI* integrase strand transfer inhibitor, *SD* standard deviation, *HIV* human immunodeficiency virus

Values are expressed as number (percentage) unless specified otherwise

One patient prescribed an entry inhibitor was excluded from this table

#### Switching patterns of anchor drug classes

Of 270 patients who switched anchor drug class from their first to their second ART regimens, two patients with multiple prescription records of anchor drug class in the second regimen were excluded (*n* = 268) [Table 4]. Most patients originally treated with NNRTI and PI switched to INSTI (91.7% [95% CI: 80.4–96.7] and 97.5% [91.4–99.3], respectively) < 1 year after starting the first regimen; 57.1% (32.6–78.6) of patients originally treated with INSTI switched to PI in the second regimen. Among patients who switched their anchor drug class ≥1 year after starting their first regimen, most originally treated with NNRTI and PI switched to INSTI (100.0% [90.1–100.0] and 97.5% [91.4–99.3], respectively), while those originally treated with INSTI equally switched to NNRTI and PI (50.0%, 21.5–78.5, for both).

**Table 4:** Switching patterns of anchor drug class in ART regimens by switching timing from 2011 to 2016

Timing of switch (*n* = 268)	Anchor drug class in the second regimen																	
	Switch < 1 year after starting the first regimen									Switch ≥1 year after starting the first regimen								
Anchor drug class in the first regimen	NNRTI			PI			INSTI			NNRTI			PI			INSTI		
	n	%	(95% CI^a^)	n	%	(95% CI^a^)	n	%	(95% CI^a^)	n	%	(95% CI^a^)	n	%	(95% CI^a^)	n	%	(95% CI^a^)
NNRTI	–	–	–	4	8.3	(3.3–19.6)	44	91.7	(80.4–96.7)	–	–	–	0	0.0	–	35	100.0	(90.1–100.0)
PI	2	2.5	(0.7–8.6)	–	–	–	79	97.5	(91.4–99.3)	2	2.5	(0.7–8.6)	–	–	–	79	97.5	(91.4–99.3)
INSTI	5	35.7	(16.3–61.2)	8	57.1	(32.6–78.6)	–	–	–	4	50.0	(21.5–78.5)	4	50.0	(21.5–78.5)	–	–	–

*ART* antiretroviral therapy, *NNRTI* non-nucleoside reverse transcriptase inhibitor, *PI* protease inhibitor, *INSTI* integrase strand transfer inhibitor, *CI* confidence interval

Two patients who had multiple prescription records of anchor drug class in the second regimen were excluded from the analysis

Entry inhibitor was excluded from the table as only two patients were prescribed an entry inhibitor in the first/second ART regimen

^a^Wilson score

#### Switching of anchor drug classes in the ART regimens

**Fig. 3:**
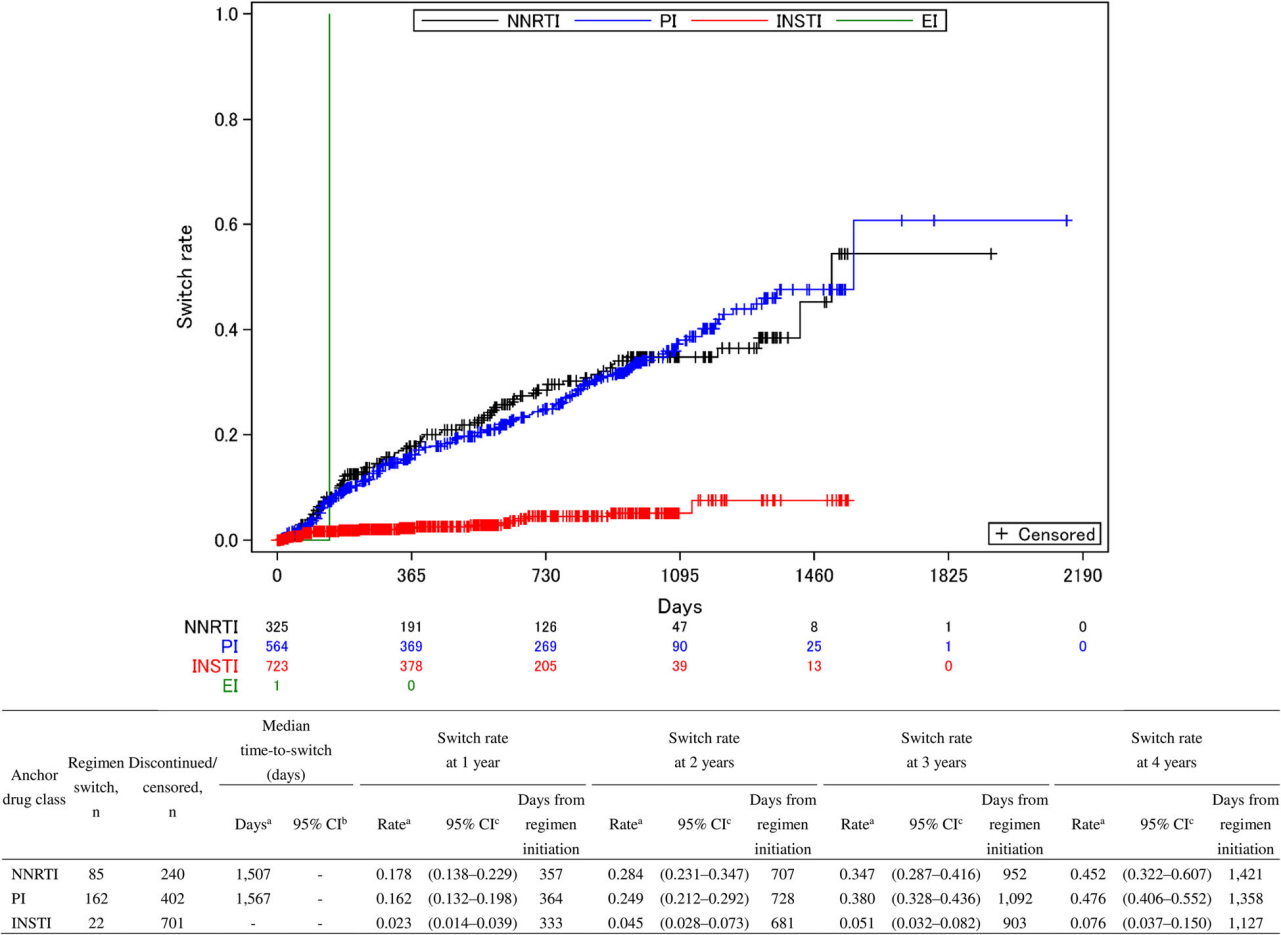
Time-to-switch of ART regimens according to anchor drug class from 2011 to 2016 (*n* = 1613). ART: antiretroviral therapy NNRTI: non-nucleoside reverse transcriptase inhibitor, PI: protease inhibitor, INSTI: integrase strand transfer inhibitor, EI: Entry inhibitor, CI: confidence interval. EI was excluded from the table as only one patient was prescribed the EI regimen. ^a^ Kaplan-Meier analysis; ^b^ Brookmeyer and Crowley method; ^c^ Greenwood method

The switch rate constantly increased over 4 years in both NNRTI (17.8–45.2%) and PI (16.2–47.6%), while INSTI maintained a low rate (2.3–7.6%) [Fig. 3]. The median time-to-switch were 1507 and 1567 days for NNRTI and PI, respectively, while that of INSTI could not be obtained due to the low regimen switch rate of 7.6% at 4 years. The results of log-rank tests indicated a significant difference in the switch rates at years one through four for any pairs of anchor drug classes (*p* < 0.05, for all), except for the comparison between NNRTI and PI at years 1, 2, and 3 (*p* = 0.398, 0.551, and 0.077, respectively).

#### Switching of anchor drug class-based backbones in the ART regimens

**Fig. 4:**
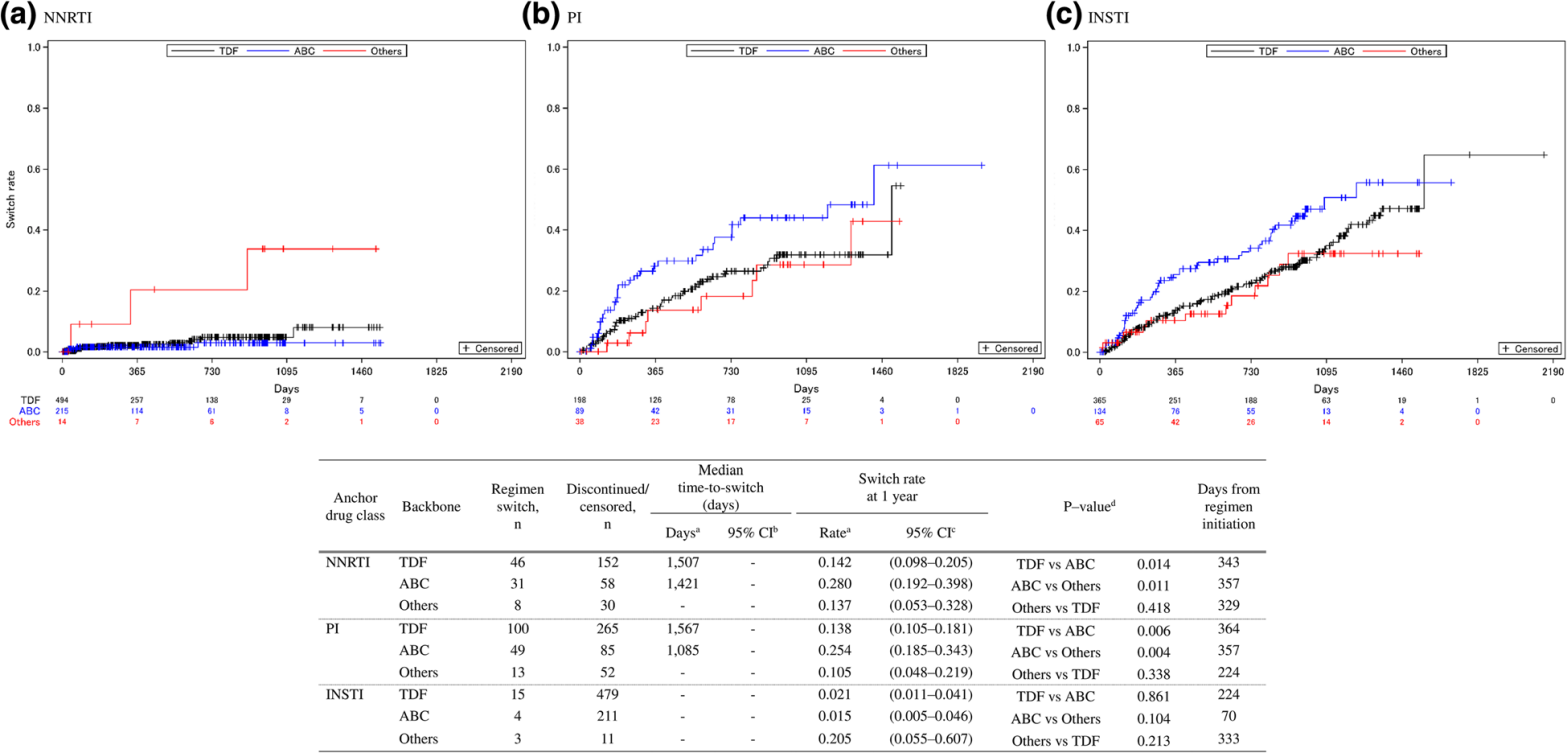
Time-to-switch of ART regimens by anchor drug class-based backbone type from 2011 to 2016 (*n* = 1613). ART: antiretroviral therapy, NNRTI: non-nucleoside reverse transcriptase inhibitor, PI: protease inhibitor, INSTI: integrase strand transfer inhibitor, ABC: abacavir, TDF: tenofovir disoproxil fumarate, CI: confidence interval. Entry inhibitor (EI) was excluded from the table as only one patient was prescribed the EI regimen. ^a^ Kaplan-Meier analysis; ^b^ Brookmeyer and Crowley method; ^c^ Greenwood method; ^d^ Log-rank test

In both NNRTI and PI, the switch rates at 1 year varied between backbones, with the lowest rate with other backbones (13.7 and 10.5%, respectively), followed by TDF (14.2 and 13.8%, respectively) and ABC (28.0 and 25.4%, respectively) [Fig. 4]. The switching rates differed significantly between TDF and ABC and between ABC and other backbones (*p* < 0.05, for all). Contrarily, in INSTI, the equally low switch rate at 1 year was observed for both ABC (1.5%) and TDF (2.1%) whereas that for other backbones was higher (20.5%). However, only a small number of patients were prescribed other backbones in any anchor drug class.

#### Assessment of potential confounding factors associated with regimen switch

**Fig. 5:**
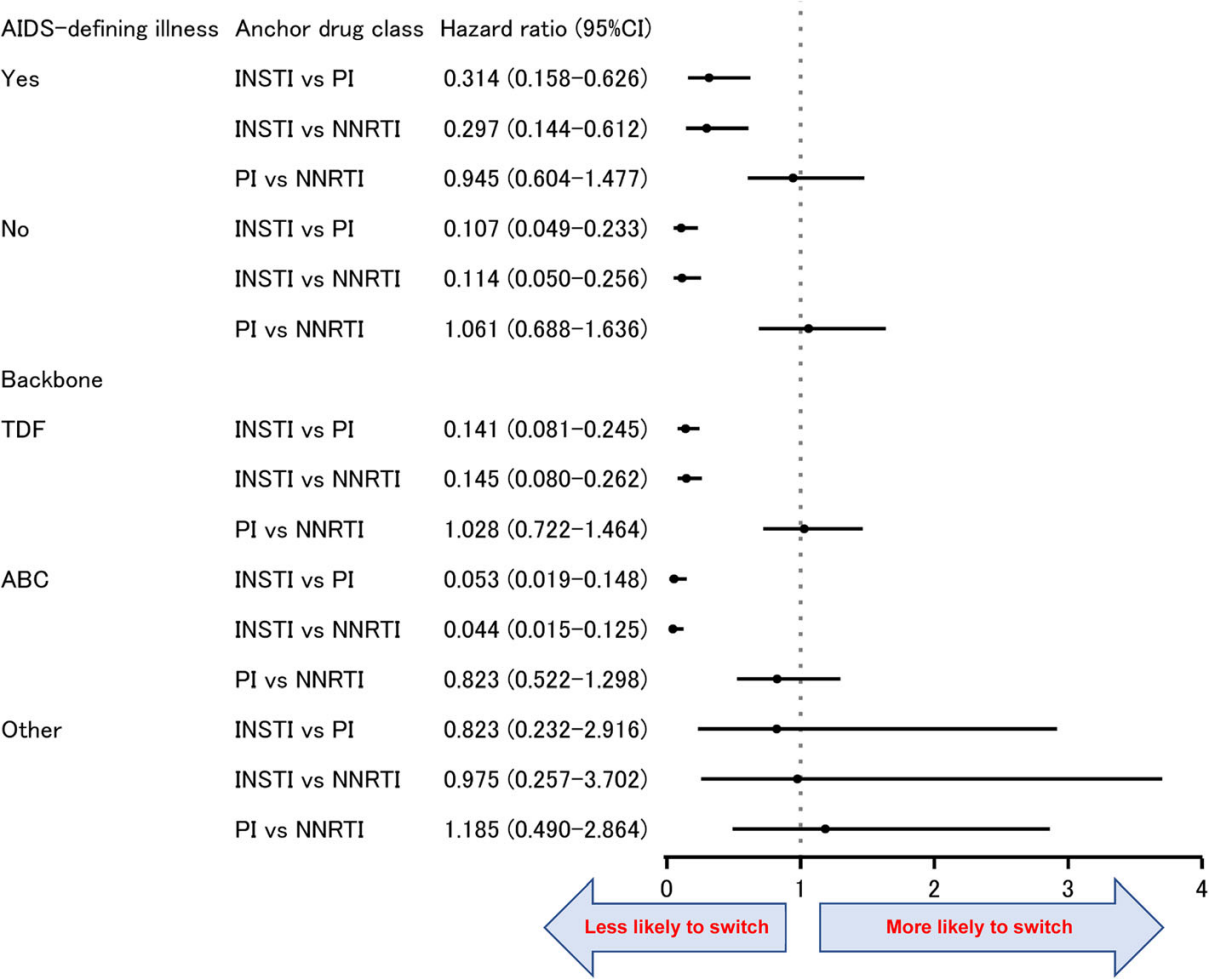
Hazard ratio for switching of each anchor drug class stratified by backbone and AIDS-defining illness. NNRTI: non-nucleoside reverse transcriptase inhibitor, PI: protease inhibitor, INSTI: integrase strand transfer inhibitor, TDF: tenofovir disoproxil fumarate, ABC: abacavir, CI: confidence interval. Six variables, including the type of backbone, anchor drug class, AIDS-defining illness, initiation year (2011–2013 or 2014–2016), and two interaction terms (between anchor drug class and AIDS-defining illness and between anchor drug class and type of backbone) were selected in the Cox regression. Subsequently, the HR was calculated for anchor drug class stratified by each interaction terms (AIDS-defining illness and type of backbone). The filled circles indicate the hazard ratio, while the horizontal lines indicate the 95% CI

In Cox regression analysis, the following four risk factors and two interaction terms were selected: AIDS-defining illness, type of backbone, year of first ART regimen (2011–2013, 2014–2016), anchor drug class, and interaction terms between anchor drug class and AIDS-defining illness and between anchor drug class and type of backbone. Subsequently, these six factors were included in the model, and HRs were calculated for anchor drug class stratified by each interaction term with anchor drug class (AIDS-defining illness and type of backbones). The HRs were constantly lower in INSTI compared with those of PI and NNRTI, regardless of the presence of AIDS-defining illness (HRs: 0.107–0.314) or type of backbone (HRs: 0.141–0.145 for TDF and 0.044–0.053 for ABC), whereas HRs for PI compared to those of NNRTI were close to 1 [Fig. 5].

## Discussion

To our knowledge, this is the first study to assess the switch rates and time-to-switch of anchor drug classes of ART regimens and the switching patterns of the anchor drug classes in these regimens, using a large hospital-based medical claims database in Japan. INSTI was the anchor drug class prescribed continuously after the first ART regimen prescribed. The common switching pattern of anchor drug class was from NNRTI or PI to INSTI. Consistent with the assessment of switch rate, the risk of switching anchor drug classes, stratified by factors with possible interaction effects with anchor drug class, was lower for patients originally prescribed INSTI than that for other anchor drug classes after adjusting for potential confounding factors.

Our results showed an increasing trend of INSTI prescriptions as an anchor drug class and a predominant prescription of TDF comprising approximately two-thirds of backbone prescriptions as the first regimen in recent years. This finding was partially consistent with the trend in initial ART regimen reported in the 2017 HIV treatment guidelines published in Japan indicating an increasing prescription of INSTI (e.g., raltegravir and dolutegravir [DTG]) as anchor drug classes in the initial regimen between 2012 and 2016 and a predominant prescription of TDF as backbone followed by ABC between 2012 and 2016, with a decreased share of TDF in 2016, perhaps due to the DTG/ABC/3TC launch in 2015 [11].

A constant increase in the switch rates of anchor drug classes over 4 years for NNRTI and PI was observed in this study, consistent with previous studies. The switch of anchor drug class over 3 years in Europe and the United States increased to over 20% among treatment-naïve PLWH treated initially with both NNRTI and PI [10]. Similarly, an increased cumulative probability of anchor drug modification in 1 year from the initiation ART regimen by 19% in efavirenz (EFV) and 23% in atazanavir/ritonavir (ATV/r) [12]. Contrarily, the switch rate in INSTI was as low as approximately 8% over 4 years and it was persistently low regardless of the type of backbone prescribed. Furthermore, the two most common switching strategies were from NNRTI or PI to INSTI regardless of switching timing among those who switched anchor drug classes from the first regimen. These findings suggest that INSTI may be the most durable anchor drug class for PLWH on ART regimens in real-world clinical-settings irrespective of backbone in the first ART regimen. These imply that the selection of an INSTI anchor drug might overcome backbone disadvantages and support a longer continuation of the initiated ART. However, it should be noted that little has been known about the continuation of INSTI in ART regimens due to a shorter follow-up time for INSTI than those of NNRTI and PI in the present study.

The prevalence of comorbidities varied among all patients on ART regimens and those who experienced an anchor drug class switch: AIDS-defining illnesses, dyslipidemia, and diabetes were more prevalent in those treated with NNRTI and PI, AIDS-defining illnesses prevailed in those originally treated with INSTI, and dyslipidemia and diabetes prevailed in those originally treated with NNRTI among those with an anchor drug class switch. These results indicated that even after adjusting for potential confounding factors and after anchor drug class was stratified according to factors with interaction effects associated with anchor drug class in multivariate analysis, the risk of switching anchor drug classes was lower in patients prescribed INSTI regardless of the presence of AIDS-defining illness or type of backbone prescribed. This finding supports the longer treatment continuation in PLWH prescribed INSTI.

We further conducted a sensitivity analysis because our results may have included patients who had initiated ART regimens before being entered into the MDV database. We included only patients who had a record of a first ART prescription between 90 to 365 days from the first visit record in the MDV database. This inclusion should have limited our patient cohort to a more realistic selection of ART-naïve patients and overcome the limitation resulting from a lack of patient information before being entered into the MDV database as patients usually start an initial ART regimen after their definitive diagnosis of HIV and their application to the social security system specific to PLWH has been completed, which may require several months from the first hospital visit. This massively reduced the number of patients included in the analysis, with few patients on PI (*n* = 31) and NNRTI (*n* = 6) and sufficient patients on INSTI (*n* = 96), suggesting that most naïve PLWH receive an INSTI class-based regimen as recommended in the current guidelines in Japan. The Kaplan-Meier curve for INSTI, however, was similar to the one depicted in Fig. 3, with similar switch rates of 1.1% (95% CI: 0.2–7.5) at 1 year and 6.0% (1.8–18.5) at years two through four. Contrarily, the curves for PI and NNRTI varied from those in Fig. 3, but because of the low number of patients, their statistical correctness is questionable. These curves hinder our interpretations owing to the insufficient number of patients on PI and NNRTI based on our definition; however, we believe that this approach validates at least our data on INSTI in our initial analysis.

This study had several limitations. First, our results may not be generalizable to PLWH in Japan. Patients who receive medical care at these hospitals may have more complicated comorbid conditions than others because hospitals with advanced medical care capabilities are included in the database. Doctors’ choices of ART regimens or switching strategies may also differ not only among hospitals in the database but also between these hospitals and HIV-specialized facilities. Second, as data included in the database were originally recorded for billing purposes, like other claims-based studies, data unavailability, including the reasons for regimen selection, reasons for regimen change/discontinuation (e.g., treatment failure, adverse events, or poor adherence), or adherence to ART regimens, limits further interpretation of our results. Third, the limited duration following INSTI introduction to Japan (July 2008) may have influenced our results as stated earlier. In fact, a small number of patients were on INSTI during our early study period (2008–2010). Further long-term study is needed to assess INSTI treatment duration. Fourth, as the database is hospital-based, the complete medical history of a patient may not be captured as stated earlier. Regardless of this limitation, our sensitivity analysis validated our results for INSTI, but due to the low numbers of patients on PI and NNRTI, the comparison requires validation with a different set of data, ideally prospective data. Finally, reporting AEs and/or asking physicians to change the regimen is partially attributable to patient engagement, which may be highly influenced by cultural barriers. Thus, the interpretation of the results of the present study may be limited to Japanese clinical-settings.

## Conclusions

In conclusion, patients with an ART regimen including INSTI as an anchor drug class maintained a low switch rate for long durations. This appeared to be independent of the presence of AIDS-defining illness or type of backbone prescribed. The major switching strategies of anchor drug class for secondary treatment were from NNRTI or PI to INSTI. These results suggest that INSTI may be a durable anchor drug class for PLWH on ART regimens. However, we cannot draw conclusions in regard to comparisons with other classes due to database limitations. Therefore, additional research on treatment duration and the reasons for switch/discontinuation is recommended to comprehend current HIV treatment in Japan.

## Additional file

Additional file 1: Type of backbone classified as others in the prescription of the first ART regimen (*n* = 118). Detailed type of backbone(s) classified into others in Fig. 2(b) (PDF 88 kb)

## Data Availability

The dataset that support the findings of this study are available from MDV, a commercial database provider. The dataset analyzed for this study cannot be shared publicly due to contractual agreements between MDV and medical facilities. For inquiries about access to the dataset used in this study, please contact MDV (website, https://www.mdv.co.jp/; e-mail, ebm_sales@mdv.co.jp).

## Abbreviations

3TC: Lamivudine
ABC: Abacavir
AE: Adverse event
ART: Antiretroviral therapy
ATV/r: Atazanavir/ritonavir
AZT: Zidovudine
CI: Confidence interval
DTG: Dolutegravir
EFV: Efavirenz
EI: Entry inhibitor
FTC: Emtricitabine
HIV: Human immunodeficiency virus
HR: Hazard ratio
ICD-10: The International Statistical Classification of Diseases and Related Health Problems 10th Revision
INSTI: Integrase strand transfer inhibitor
NNRTI: Non-nucleoside reverse transcriptase inhibitor
NRTI: Nucleoside reverse transcriptase inhibitor
PI: Protease inhibitor
PLWH: People living with human immunodeficiency virus
TAF: Tenofovir alafenamide fumarate
TDF: Tenofovir disoproxil fumarate

## References

[1] Antiretroviral Therapy Cohort Collaboration (2008). Life expectancy of individuals on combination antiretroviral therapy in high-income countries: a collaborative analysis of 14 cohort studies. Lancet.

[2] Costagliola D (2014). Demographics of HIV and aging. Curr Opin HIV AIDS.

[3] Samji H, Cescon A, Hogg RS, Modur SP, Althoff KN, Buchacz K (2013). North American AIDS cohort collaboration on research and design (NA-ACCORD) of IeDEA. Closing the gap: increases in life expectancy among treated HIV-positive individuals in the United States and Canada. PLoS One.

[4] Nakagawa F, May M, Phillips A (2013). Life expectancy living with HIV: recent estimates and future implications. Curr Opin Infect Dis.

[5] van Sighem AI, Gras LA, Reiss P, Brinkman K, de Wolf F (2010). ATHENA national observational cohort study. Life expectancy of recently diagnosed asymptomatic HIV-infected patients approaches that of uninfected individuals. AIDS.

[6] DHHS panel on antiretroviral guidelines for adults and adolescents. Guidelines for the use of antiretroviral agents in HIV-1-infected adults and adolescents. Updated on October 25, 2018. Available from: https://aidsinfo.nih.gov/contentfiles/lvguidelines/adultandadolescentgl.pdf. Accessed 3 June 2019.

[7] The Guidelines for the Treatment of HIV Infection, March 2019 version. Japanese Ministry of Health, Labour and Welfare. 1–156. Available from: https://www.haart-support.jp/guideline.htm. Accessed 3 June 2019.

[8] Elzi L, Marzolini C, Furrer H, Ledergerber B, Cavassini M, Hirschel B (2010). Swiss HIV cohort study. Treatment modification in human immunodeficiency virus-infected individuals starting combination antiretroviral therapy between 2005 and 2008. Arch Intern Med.

[9] Prosperi MC, Fabbiani M, Fanti I, Zaccarelli M, Colafigli M (2012). Predictors of first-line antiretroviral therapy discontinuation due to drug-related adverse events in HIV-infected patients: a retrospective cohort study. BMC Infect Dis.

[10] Abgrall S, Ingle SM, May MT, Costagliola D, Mercie P, Cavassini M (2013). Antiretroviral therapy cohort collaboration (ART-CC). Durability of first ART regimen and risk factors for modification, interruption or death in HIV-positive patients starting ART in Europe and North America 2002–2009. AIDS.

[11] The Guidelines for the Treatment of HIV Infection, March 2017 version. The Japanese Ministry of Health, Labour and Welfare. 1–152.

[12] Jarrin I, Hernández-Novoa B, Alejos B, Santos I, Lopez-Aldeguer J, Riera M (2014). Cohort of the Spanish HIV research network (CoRIS). Interpreting the reasons for the choice and changing of two drug regimens in an observational cohort: comparison of a ritonavir-boosted protease inhibitor-based versus a nonnucleoside reverse transcriptase inhibitor-based first-line regimen. HIV Med.

