# Supplementary material for: Switch rates, time-to-switch, and switch patterns of antiretroviral therapy in people living with human immunodeficiency virus in Japan, in a hospital-claim database
Source: BMC Infect Dis. 2019 Jun 10;19:505. doi: 10.1186/s12879-019-4129-6 (PMC6558763; doi:10.1186/s12879-019-4129-6)
Supplement: Supplementary file 1 — Type of backbone classified as others in the prescription of the first ART regimen (n = 118). Detailed type of backbone(s) classified into others in Fig. 2(b) (PDF 88 kb) [file 12879_2019_4129_MOESM1_ESM.pdf]

Additional file 1. Type of backbone classified as others in the prescription of the first ART regimen (n = 118)

Backbone(s)	Anchor drug class prescribed in the first ART regimen			
	Overall	NNRTI (n = 85)	PI (n = 162)	INSTI (n = 22)
AZT	1 (0.8)	0 (0.0)	0 (0.0)	1 (7.1)
3TC	3 (2.5)	2 (5.3)	1 (1.5)	0 (0.0)
d4T	1 (0.8)	0 (0.0)	1 (1.5)	0 (0.0)
AZT/3TC	44 (37.3)	16 (42.1)	23 (35.4)	5 (35.7)
3TC and AZT	43 (36.4)	11 (28.9)	30 (46.2)	2 (14.3)
3TC and d4T	17 (14.4)	7 (18.4)	9 (13.8)	1 (7.1)
ABC and TDF/FTC	1 (0.8)	1 (2.6)	0 (0.0)	0 (0.0)
ddl and d4T	1 (0.8)	1 (2.6)	0 (0.0)	0 (0.0)
No backbone	7 (5.9)*	0 (0.0)	1 (1.5)	5 (35.7)

ART: antiretroviral therapy, NNRTI: non-nucleoside reverse transcriptase inhibitor, PI: protease inhibitor, INSTI: integrase strand transfer inhibitor, AZT: zidovudine, 3TC: lamivudine, d4T: 2′,3′-didehydro-3′-deoxythymidine, ABC: abacavir, TDF: tenofovir disoproxil fumarate, FTC: emtricitabine, ddl: didanosine

*One patient prescribed an entry inhibitor was included.
